# The Nlrp3 Inflammasome Orchestrates Mobilization of Bone Marrow-Residing Stem Cells into Peripheral Blood

**DOI:** 10.1007/s12015-019-09890-7

**Published:** 2019-05-14

**Authors:** Anna M. Lenkiewicz, Mateusz Adamiak, Arjun Thapa, Kamila Bujko, Daniel Pedziwiatr, Ahmed K. Abdel-Latif, Magda Kucia, Janina Ratajczak, Mariusz Z. Ratajczak

**Affiliations:** 1 Center for Preclinical Studies and Technology, Department of Regenerative Medicine at Medical University of Warsaw, Warsaw, Poland; 2 Stem Cell Institute at James Graham Brown Cancer Center, University of Louisville, 500 S. Floyd Street, Rm. 107, Louisville, KY 40202 USA; 3 Division of Cardiovascular Medicine, Gill Heart Institute, University of Kentucky, Lexington, KY USA

**Keywords:** Nlrp3 Inflammasome, Purinergic signaling, Extracellular nucleotides, Complement cascade, Stem cell mobilization

## Abstract

Mobilization of stem cells from bone marrow (BM) into peripheral blood (PB) in response to tissue or organ injury, infections, strenuous exercise, or mobilization-inducing drugs is as we postulated result of a “sterile inflammation” in the BM microenvironment that triggers activation of the Complement Cascade (ComC). Therefore, we became interested in the role of the Nlrp3 inflammasome in this process and show for the first time that its activation in ATP-dependent manner orchestrates BM egress of hematopoietic stem/progenitor cells (HSPCs) as well as other stem cells, including mesenchymal stroma cells (MSCs), endothelial progenitor cells (EPCs), and very small embryonic-like stem cells (VSELs). To explain this extracellular ATP is a potent activator of the Nrlp3 inflammasome, which leads to the release of interleukin 1β and interleukin 18, as well as several danger-associated molecular pattern molecules (DAMPs) that activate the mannan-binding lectin (MBL) pathway of the ComC, from cells of the innate immunity network. In support of this mechanism, we demonstrate that the Nlrp3 inflammasome become activated in innate immunity cells by granulocyte colony stimulating factor (G-CSF) and AMD3100 in an ATP-dependent manner. Moreover, administration of the Nlrp3 inflammasome activator nigericin induces mobilization in mice, and the opposite effect is obtained by administration of an Nlrp3 inhibitor (MCC950) to mice mobilized by G-CSF or AMD3100. In summary, our results further support the crucial role of innate immunity, BM sterile inflammation, and novel role of the ATP–Nlrp3–ComC axis in the egress of stem cells into PB.

## Introduction

Hematopoietic stem/progenitor cells (HSPCs) as well as other stem/progenitor cells residing in bone marrow (BM), such as mesenchymal stroma cells (MSCs), endothelial progenitors (EPCs), and very small embryonic-like stem cells (VSELs), are mobilized into peripheral blood (PB) in response to infection, stress, tissue or organ injury, or pharmacological mobilization after administration of pro-mobilizing drugs [1–5]. Both cytokine granulocyte colony stimulating factor (G-CSF) as well as AMD3100, which is a small molecule that blocks the CXCR4 receptor, are employed to mobilize HSPCs that are subsequently employed for hematopoietic transplantation [6–10]. This pharmacologically induced mobilization increases the number of HSPCs circulating in PB by up to 100 fold. However, the mechanisms that regulate egress of HSPCs into PB are still not well understood, and because of the importance of stem cell trafficking in maintaining organism homeostasis, several complementary pathways are believed to be involved [6–10].

We previously proposed that mobilization of HSPCs is mainly the result of “sterile” inflammation in the BM microenvironment in response to pro-mobilizing stimuli, leading to activation of BM-residing cells belonging to the innate immunity network, including granulocytes and monocytes [11, 12]. In response to G-CSF or AMD3100, these cells i) release proteolytic and lipolytic enzymes that free HSPCs from their BM niches and ii) secrete danger-associated molecular pattern molecules (DAMPs, also known as alarmins) that activate the mannan-binding lectin (MBL) pathway of Complement Cascade (ComC) activation.^13^ The most important DAMP activators of the MBL pathway are high molecular group box 1 (Hmgb1) and S100 calcium-binding protein A9 (S100a9) [13, 14].

An important role in the release of HSPCs is played by extracellular nucleotides, and in particular, adenosine triphosphate (ATP), which after secretion from activated BM cells into the extracellular space becomes a potent activator of several purinergic receptors [13, 15, 16]. In our previous work we found that inhibition of ATP secretion in BM, by blocking its release into the extracellular space or by eliminating one of its receptors (P2X7) on the surface of hematopoietic cells, leads to a decrease in mobilization efficiency [13]. Based on evidence that the ATP–P2X7 receptor interaction leads to activation of the Nrlp3 inflammasome in several types of cells, including cells belonging to the innate immunity network [17–20], we asked whether this inflammasome is required for optimal ATP-induced mobilization of HSPCs.

To address this question, we analyzed the expression of Nrlp3 elements at the mRNA and protein levels after G-CSF and AMD3100 administration in mice, employed Nrlp3 inflammasome activators and small-molecule inhibitors, and finally confirmed our observation in Nlrp3-KO mice. Based on these results, we propose a crucial role for BM sterile inflammation and involvement of the ATP–Nlrp3 inflammasome–ComC axis in the egress of stem cells into PB. These results will be important in developing more efficient clinical stem cell mobilization strategies.

## Material and Methods

### Animals

Pathogen-free, 4–6-week-old C57BL/6 J wild-type (WT) mice were bred at the University of Louisville or purchased from the Jackson Laboratory (Bar Harbor, ME, USA) at least 2 weeks prior to experiments. Animal studies were approved by the Animal Care and Use Committee of the University of Louisville (Louisville, KY, USA).

### In Vivo Mobilization Studies

Mice were mobilized with G-CSF (Amgen, Thousand Oaks, CA, USA) once or over 3 days at 100 μg/kg/day by subcutaneous injection (SC) or with AMD3100 (Sigma-Aldrich, St. Louis, MO, USA) for 1 day at 5 mg/kg by intraperitoneal injection (IP). In some cases mice received IL-1 (1 μg/mouse), IL-18 (1 μg/mouse), nigericin (0.5 or 1 mg/kg), HMGB1 (1.25 μg/mouse), or the inflammasome inhibitor MCC950 (50 μg/kg). Depending on the experiment, at 1, 6, or 24 h after the last G-CSF injection, 1 h after AMD3100 injection, 6 h after lL-1 or Il-18 injection, and 1 or 4 h or 3 days after nigericin injection, the mice were bled from the retro-orbital plexus to obtain plasma for genomic and proteomic experiments and hematology analysis, while PB was obtained from the vena cava (with a 25-gauge needle and 1-ml syringe containing 250 U heparin). Mononuclear cells (MNCs) were obtained by hypotonic lysis of RBCs in BD Pharm Lyse buffer (BD Biosciences), as described [13].

### Evaluation of HSPC Mobilization

For evaluation of circulating colony-forming unit-granulocyte/macrophage (CFU-GM) and SKL cells, the following formulas were used: (number of white blood cells [WBCs] × number of CFU-GM colonies)/number of WBCs plated = number of CFU-GM per ml of PB; and (number of WBCs × number of SKL cells)/number of gated WBCs = number of SKL cells per μl of PB [13, 21].

### PB Parameter Counts

To obtain white blood cell counts, 50 μl of PB was taken from the retro-orbital plexus of mice into microvette EDTA-coated tubes (Sarstedt Inc., Newton, NC, USA) and run on a HemaVet 950FS hematology analyzer (Drew Scientific Inc., Oxford, CT, USA) within 2 h of collection [13, 21].

### Clonogenic CFU-GM Assay

Peripheral blood mononuclear cells (PBMNCs, 1 × 10^6^) were resuspended in human methylcellulose base medium (R&D Systems, Minneapolis, MN, USA), supplemented with 25 ng/ml recombinant murine granulocyte/macrophage colony-stimulating factor (mGM-CSF; PeproTech, Rocky Hill, NJ, USA) and 10 ng/ml recombinant murine interleukin 3 (mIL-3; PeproTech). Cells were incubated for 7–14 days (37 °C, 95% humidity, and 5% CO_2_), and the numbers of CFU-GM colonies were scored using an inverted microscope (Olympus, Center Valley, PA, USA). Final results were recalculated based on the number of PBMNCs/μl of PB, as described above [13, 21].

### Fluorescence-Activated Cell Sorting (FACS) Analysis

For staining of Lin^−^/Sca-1^+^/c-Kit^+^ (SKL cells), Sca-1^+^/Lin^−^/CD45^−^ (VSELs), Lin^−^/CD45^−^/CD31^+^ (EPCs), and Lin^−^/CD45^−^/CD31^−^/CD90^+^ (MSCs), the following monoclonal antibodies were used: FITC–anti-CD117 (also known as c-Kit, clone 2B8; BioLegend, San Diego, CA, USA) and PE–Cy5–anti-mouse Ly-6 A/E (also known as Sca-1, clone D7; eBioscience, San Diego, CA, USA). All anti-mouse lineage marker antibodies, including anti-CD45R (also known as B220, clone RA3-6B2), anti-Ter-119 (clone TER-119), anti-CD11b (clone M1/70), anti-T cell receptor β (clone H57–597), anti-Gr-1 (clone RB6-8C5), anti-TCRγδ (clone GL3), and anti-CD45 (clone 30-F11), conjugated with PE; anti-CD31 (clone MEC 13.3), conjugated with APC; and anti-CD90.2 (clone 30-H12), conjugated with BV510, were purchased from BD Biosciences. Staining was performed in RPMI-1640 medium containing 2% FBS. All monoclonal antibodies were added at saturating concentrations, and the cells were incubated for 30 min on ice, washed twice, and analyzed with an LSR II flow cytometer (BD Biosciences) [21].

### Isolation of gr-1^+^/CD11b^+^ Cells

Gr-1^+^/CD11b^+^ cells were isolated from the BM of adult mice as described [22]. Briefly, the BM was flushed from femurs, and the population of total nucleated cells was obtained after lysis of red blood cells (RBCs) using 1 × BD Pharm Lyse buffer (BD Pharmingen, San Jose, CA, USA). The cells were subsequently stained with phycoerythrin (PE)–anti-Gr-1 antibody (anti-Ly-6G and Ly-6C, clone RB6-8C5) and PE–anti-CD11b antibody (clone M1/70) for 30 min in medium containing 2% fetal bovine serum (FBS). The cells were then washed, resuspended in RPMI-1640 medium, and sorted as populations of granulocytes using a Moflo XDP cell sorter (Beckman Coulter, Indianapolis, IN, USA).

### Real-Time Quantitative Polymerase Chain Reaction to Evaluate the Expression of Inflammasome Genes

Total bone marrow peripheral blood RNA or RNA isolated from Gr-1^+^/CD11b^+^ cells was isolated using the RNeasy Mini Kit (Qiagen Inc., Valencia, CA, USA), while messenger RNA was reverse transcribed with iScript (Bio-Rad). The resulting cDNA fragments were amplified using the SYBR Green system (Applied Biosystems, Carlsbad, CA, USA). Primer sequences for the genes encoding β2 microglobulin (β2m), Nlrp3, Asc, caspase 1, interleukin 1β, interleukin 18, Hmgb-1, and S100a9 (calgranulin B) are as follows:

- β2m
* forward primer: 5′-ATGCTATCCAGAAAACCCCTCAAAT-3′
* reverse primer: 5′-AACTGTGTTACGTAGCAGTTCAGTA-3′
- Nlrp3
* forward primer: 5′- ACCAGCCAGAGTGGAATGAC -3′
* reverse primer: 5′- ATGGAGATGCGGGAGAGATA -3′
- Asc (also known as Pycard)
* forward primer: 5′- GCCAGAACAGGACACTTTGTG -3′
* reverse primer: 5′- AGTCAGCACACTGCCATGC -3′
- Casp1
* forward primer: 5′- GCTTTCTGCTCTTCAACACC -3′
* reverse primer: 5′- AAAATGTCCTCCAAGTCACAAG -3′
- Il-1β
* forward primer: 5′- AGTTGACGGACCCCAAAAG -3′
* reverse primer: 5′- CTTCTCCACAGCCACAATGA -3′
- Il-18
* forward primer: 5′- ACAACTTTGGCCGACTTCAC -3′
* reverse primer: 5′- GTCTGGTCTGGGGTTCACTG -3′
- Hmgb1
* forward primer: 5′- GGAGGAGCACAAGAAGAAGC -3′
* reverse primer: 5′- GGGGGATGTAGGTTTTCATTT -3′
- S100a9
* forward primer: 5′- TGGTGGAAGCACAGTTGG -3′
* reverse primer: 5′- CATCAGCATCATACACTCCTCAA -3′

The relative value of the target, normalized to an endogenous control gene (β2m) and relative to a calibrator, is expressed as 2^–ΔΔCt^ (fold difference), in which ΔCt equals the Ct of the target gene minus the Ct of the endogenous control gene (β2m), and ΔΔCt equals the ΔCt of the samples for the target gene minus the ΔCt of the calibrator for the target gene. To avoid the possibility of amplifying DNA contamination, uniform amplification of the products was rechecked by analyzing the melting curves of the amplified products (dissociation curves). It was found that the melting temperature (Tm) was 57–60 °C, while the product Tm was at least 10 °C higher than the primer Tm.

### Enzyme-Linked Immunosorbent Assay for IL-1β, IL-18, and Hmgb-1 Detection

Whole blood from mice was obtained from the vena cava (1-ml syringe containing 100 μl of 0.5 M EDTA). Plasma samples were prepared by taking the top fraction after centrifugation at 600×g for 10 min at 4 °C and immediately freezing at −80 °C. Plasma samples were used at a 1:4 dilution (with sterile PBS). The residual IL-1β, IL-18, and Hmgb-1 levels were measured by enzyme-linked immunosorbent assay (ELISA) according to the manufacturer’s protocols (IL-1β, Cloud-Clone cat. no. SEA563Mu; IL-18, Affymetrix eBioscience cat. no. BMS618/3; Hmgb-1,Cloud-Clone cat. no. SEA399Mu). Results (absorbance) are presented as % of control [13, 23, 24].

### Statistical Analysis

All results are presented as mean ± SD. All results are presented as mean ± SD. Statistical analysis of the data was done using unpaired Student’s t test or one-way ANOVA followed by Dunnett’s multiple comparisons test (*p < 0.05; **p < 0.01; ***p < 0.001 compared with control).

## Results

### Activation of the Nlrp3 Inflammasome in BM-Sorted gr-1^+^ Cells

In our previous work we demonstrated the pivotal role of extracellular ATP and purinergic signaling in the mobilization of HSPCs [12, 13]. ATP release from activated cells was correlated with mobilization efficacy and activation of the ComC [13]. Since extracellular ATP is a potent activator of the Nlrp3 inflammasome [17–20] a major focus of the current work was to test whether the Nlrp3 inflammasome serves as a cogwheel or gear between purinergic signaling and the ComC to direct egress of HSPCs from BM into PB.

**Fig. 1:**
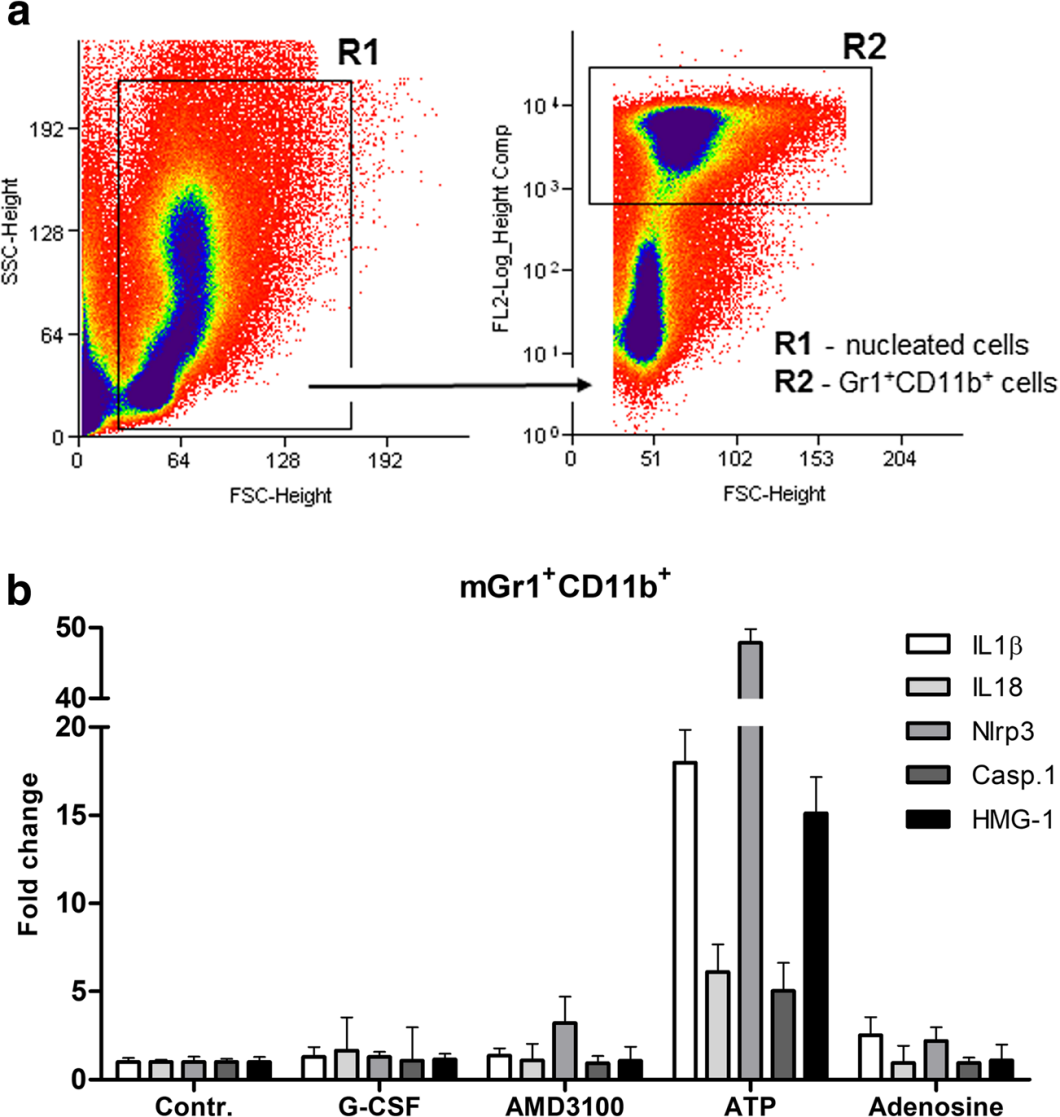
**Expression of mRNA for the Nlrp3 inflammasome complex in sorted Gr1**^**+**^**/CD11b**^**+**^**BM cells after stimulation with ATP, G-CSF, or AMD3100.** Panel **a.** Sorting strategy for BM-derived Gr-1^+^/CD11b^+^ cells. Please not that both Gr1 and CD11b were labeled with PE. Panel **b.** Sorted Gr-1^+^/CD11b^+^ cells were stimulated for 6 h in serum medium supplemented with 0.5% BSA with G-CSF, AMD3100, ATP, or the ATP metabolite adenosine. As shown, only ATP upregulated expression of mRNAs for Nlrp3 inflammasome components in Gr-1^+^/CD11b^+^ cells. Representative results from two experiments are shown. Isolation of Gr-1+/CD11b + cells. Gr-1+/CD11b + cells were isolated from the BM of adult C57BL/6 J mice as described. Briefly, BM was flushed from tibias and femurs, and the population of total nucleated cells was obtained after lysis of red blood cells (RBCs) using 1 × BD Pharm Lyse buffer (BD Pharmingen, San Jose, CA, USA). The cells were subsequently stained with antibodies (BD Biosciences, San Jose, CA, USA): phycoerythrin (PE)–anti-Gr-1 (anti-Ly-6G and Ly-6C, clone RB6-8C5) and PE-anti-CD11b (clone M1/70) for 30 min in RPMI-1640 medium containing 2% fetal bovine serum (FBS). The cells were then washed, resuspended in RPMI-1640 medium and sorted using a Moflo XDP cell sorter (Beckman Coulter, Indianapolis, IN, USA) as populations of granulocytes/monocytes (Gr-1+/CD11b+)

First, we sorted a population of Gr-1^+^/CD11b^+^ cells from murine BM (Fig. 1a), which are enriched for monocytes and granulocytes. These sorted cells were subsequently stimulated for 6 h in serum medium supplemented with 0.5% BSA with G-CSF, AMD3100, ATP, or the ATP metabolite adenosine. We found that only ATP upregulated expression of the mRNA for Nlrp3 inflammasome components in Gr-1^+^/CD11b^+^ cells (Fig. 1b). This finding indicates that, as proposed in our previous work, G-CSF and AMD3100 must first release ATP from target cells to trigger, via purinergic signaling, egress of HSPCs from BM into PB [13].

### Administration of G-CSF and AMD3100 Activates the Nlrp3 Inflammasome

**Fig. 2:**
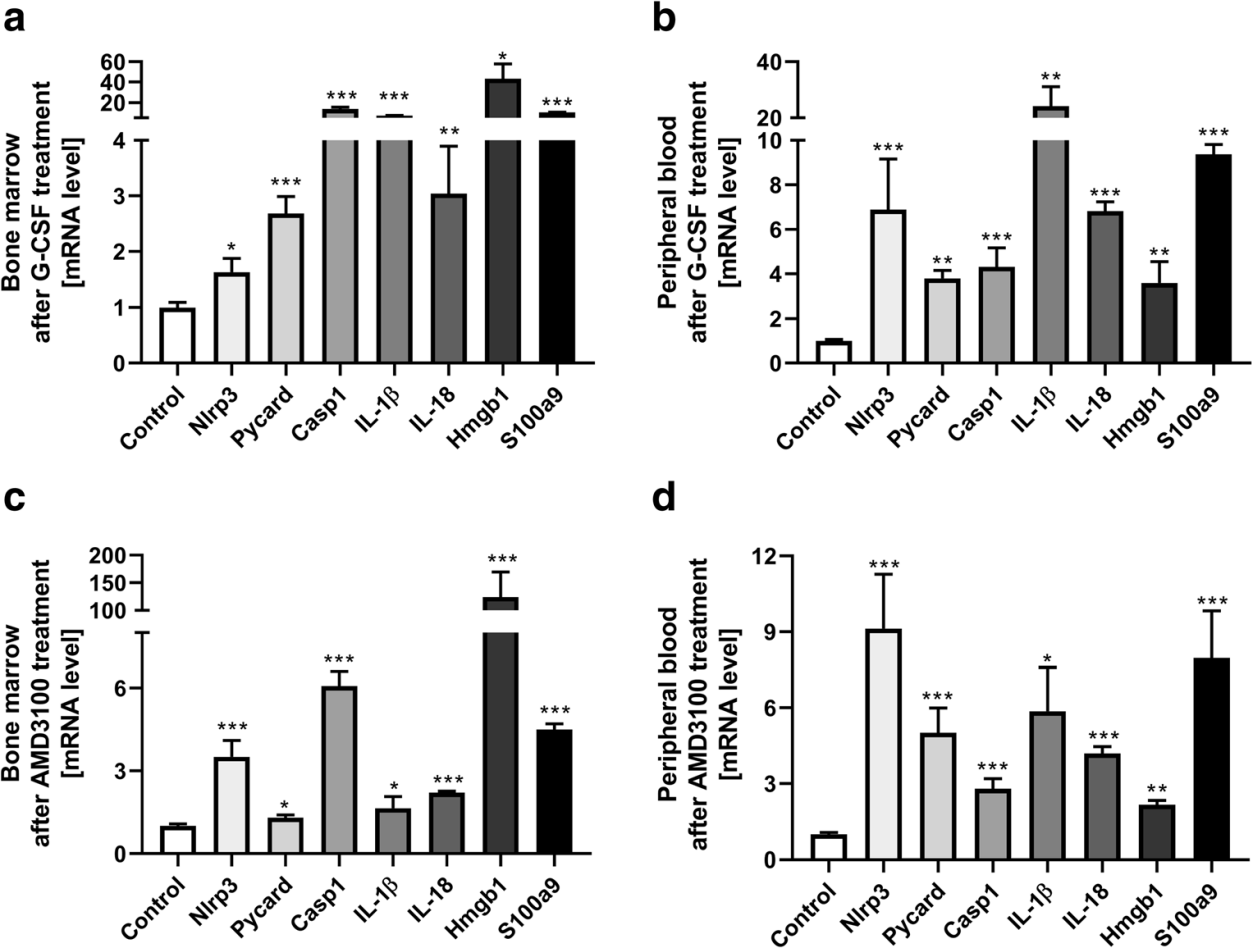

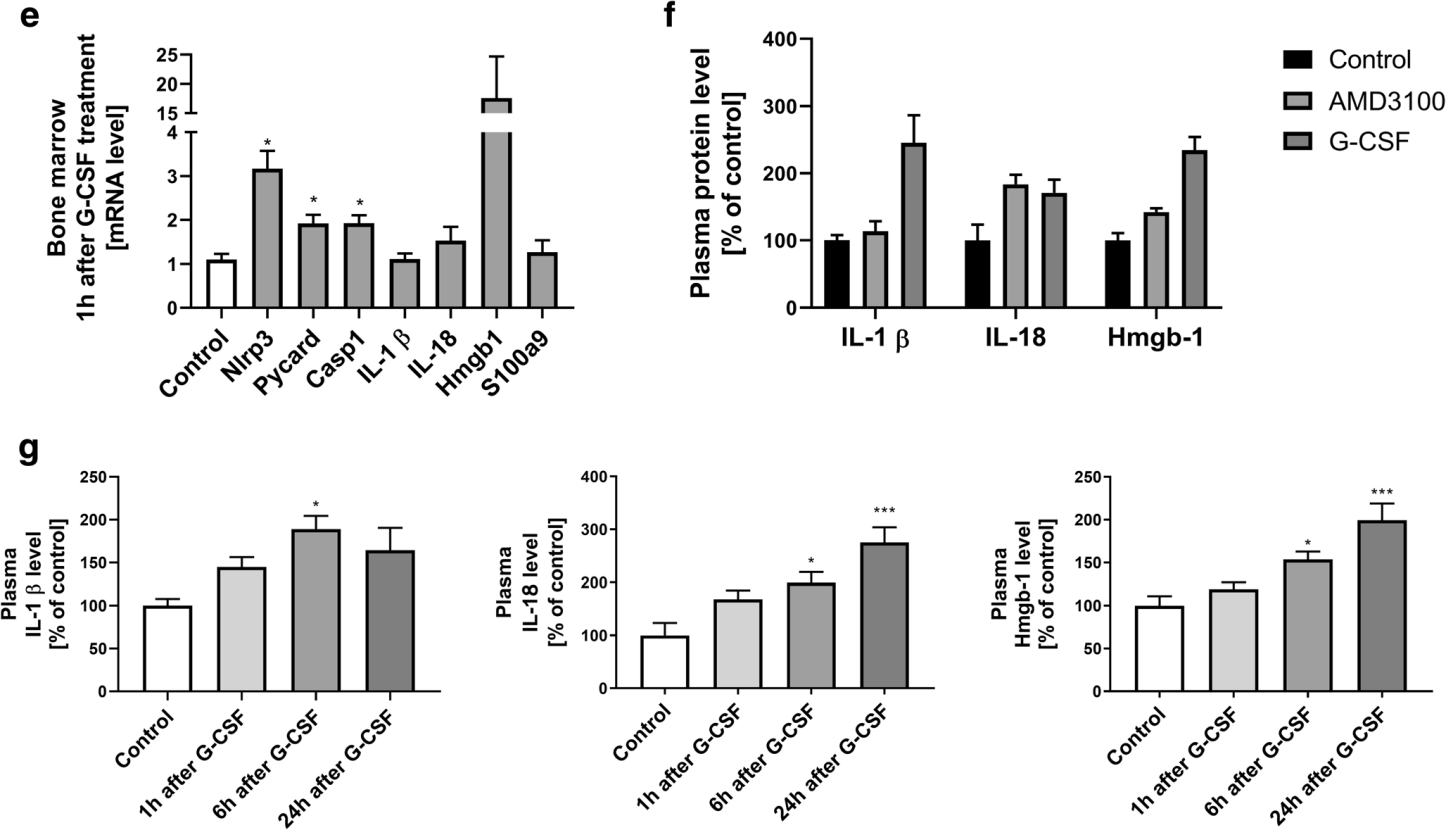
**Effect of the mobilization agents AMD3100 and G-CSF on mRNA and protein expression related to Nlrp3 inflammasome activation, as measured by qRT-PCR and ELISA**. Panel **a**. Expression of Nlrp3, Asc (Pycard), Casp1, IL-1β, Il-18, Hmgb1, and S100a9 mRNAs in bone marrow after 3-day G-CSF (100 μg/kg/day) treatment as measured by qRT-PCR. Panel **b**. Expression of the same mRNAs in peripheral blood after 3-day G-CSF (100 μg/kg/day) treatment as measured by qRT-PCR. Panel **c**. Expression of the same mRNAs in bone marrow after AMD3100 (5 mg/kg) treatment as measured by qRT-PCR. Panel **d**. Expression of the same mRNAs in peripheral blood after AMD3100 (5 mg/kg) treatment as measured by qRT-PCR. Panel **e**. Expression of the same mRNAs in bone marrow after 1-h treatment with G-CSF (100 μg/kg) as measured by qRT-PCR. Panel **e**. The level of IL-1β, IL-18 and Hmgb-1 proteins in mouse plasma after AMD3100 (5 mg/kg) or 3-day G-CSF (100 μg/kg/day) treatment as measured by ELISA. **Panel G**. The level of IL-1β, IL-18, and Hmgb-1 proteins in mouse plasma after 1, 6, and 24 h treatment with G-CSF (100 μg/kg) as measured by ELISA. The data represent the mean value ± SEM for four independent experiments. Results of qRT-PCR were normalized to the β2 microglobulin (β2m) level. Results for ELISA are presented as a percentage of control. The data represent the mean value ± SEM for four independent experiments. *p < 0.05; **p < 0.01; ***p < 0.001 compared with control (Student’s t test or one-way ANOVA followed by Dunnett’s multiple comparisons test)

To address the involvement of Nlrp3 inflammasome activation in response to G-CSF and AMD3100 administration, we evaluated the expression of genes that are involved in formation of the inflammasome complex by qRT-PCR [17–20]. Figure 2 shows changes in expression of mRNA for the genes encoding Nlrp3, Asc (Pycard), Caspase 1 (Casp1), IL-1β, IL-18, Hmgb1, and S100a9 in murine BM (Fig. 2a) and in PB (Fig. 2b**)** cells after three days of G-CSF mobilization, measured at 6 h after the last dose of G-CSF. Similarly, Fig. 2c, d shows changes in mRNA expression for Nlrp3 inflammasome components 1 h after administration of AMD3100 in BM and PB cells, respectively. Results were normalized to the β2 microglobulin level. As a result of pharmacological mobilization we observed a significant upregulation of mRNA for several genes associated with the Nlrp3 inflammasome complex.

We also analyzed mRNA expression at 1 h after single G-CSF injection and observed that mRNA for the Nlrp3 inflammasome was already upregulated at this time point (Fig. 2e). However, direct comparison of changes in expression of other components of the Nrlp3 inflammasome indicate that there are differences in the kinetics of mRNA expression for inflammasome-associated genes 1 h after administration of G-CSF versus 1 h after administration of AMD3100 (Fig. 2c). For example, expression of Casp1 and S100a9 mRNA was at a higher level of expression in BM cells exposed to AMD3100. These observed differences require further study.

Finally, as expected, ELISA-detected expression of selected protein components of the Nlrp3 inflammasome demonstrated an increase in IL-1β and IL-18 as well as Hmgb1 in PB after AMD3100 injection and 3 days of mobilization performed with G-CSF (Fig. 2f). Moreover, kinetic studies of a single administration of G-CSF (Fig. 2g) revealed that there is a time delay in upregulation of IL-1β, IL-18, and Hmgb1 at the protein level, as a significant increase in the level of these proteins occurred in PB a few hours after administration of a single dose of G-CSF.

### Activation of the Nlrp3 Inflammasome by Nigericin Mobilizes BM Stem Cells into PB

It is well known that ATP binds to the P2X7 receptor to trigger activation of the Nlrp3 inflammasome [17–20]. Specifically, the ATP–P2X7 receptor interaction increases Ca^2+^ influx, and by engaging the TWIK-2 potassium efflux channel decreases the intracellular level of K^+^ [25]. This change in intracellular K^+^ level triggers as reported activation of Nlrp3 and caspase 1 [25]. Nigericin, a microbial toxin derived from *Streptomyces hydroscopicus*, is a potassium ionophore and a potent activator of the Nlrp3 inflammasome [26]. Therefore, we employed nigericin and found that it activates the Nlrp3 inflammasome and triggers mobilization of HSPCs.

**Fig. 3:**
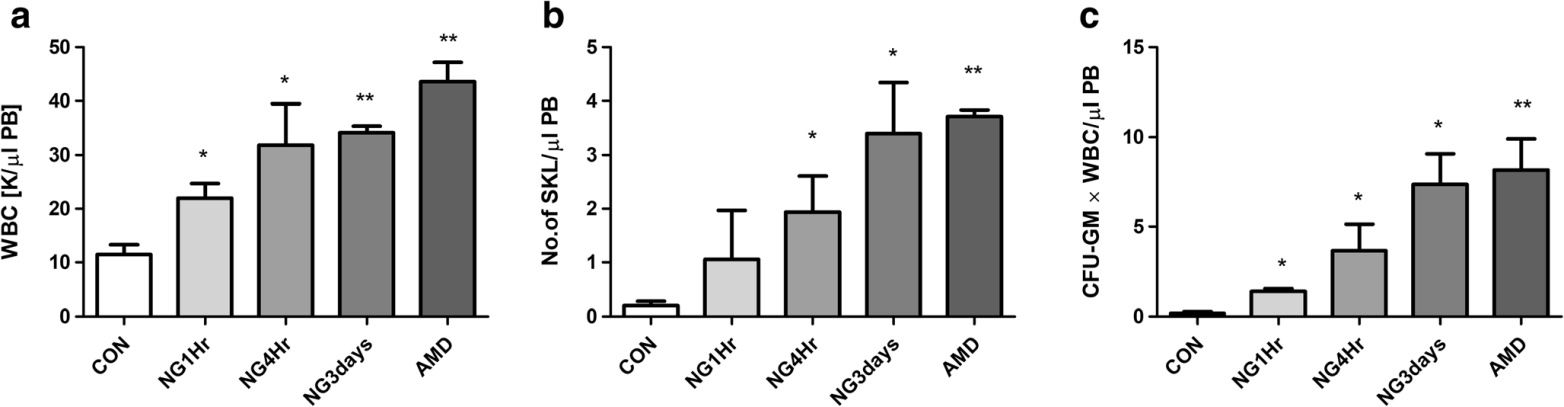
**Nigericin mobilizes HSPCs into peripheral blood.** Representative results show the PB parameters for WBCs (**a**), SKL cells (**b**), and CFU-GM clonogenic progenitors (**c**) from mice injected i.p. with PBS, AMD3100 (5 mg/kg), or nigericin (1 mg/kg or 0.5 mg/kg). AMD3100-injected mice were sacrificed 1 h post-injection. Mice that received a dose of 1 mg/kg nigericin were sacrificed 1 h (NG1hr) or 4 h (NG4hr) post-injection, whereas mice that were injected for 3 days (0.5 mg/kg) were sacrificed 4 h after the last injection (NG3days). The number of SKL cells mobilized into PB was calculated using the formula, WBCs x SKL cells/gated WBCs = SKL cells/μl; and CFU-GM/μl of PB was evaluated by the formula, [WBCs] x CFU-GM colonies/WBCs plated. The data are presented as means ± S.E. Unpaired Student’s t test was used for the determination of significance (*p ≤ 0.05, **p ≤ 0.01)

As shown in Fig. 3**,** we observed a dose-dependent effect of nigericin on HSPC mobilization. Prolonged (3-day) administration of nigericin in mice led to a release of HSPCs comparable to a single AMD3100 injection. However, we found that nigericin as a strong activator of the Nlrp3 inflammasome induces a mild systemic inflammatory reaction in experimental animals.

### Inhibition of the Nlrp3 Inflammasome by MCC950 Decreases Mobilization of BM Stem Cells

To address directly the role of the Nlrp3 inflammasome in the mobilization of BM-residing stem cells, we employed an Nlrp3 inflammasome inhibitor, the small molecule MCC950. This molecule has been demonstrated to be a specific inhibitor of the Nlrp3 inflammasome by preventing its interaction with the ASC (Pycard) protein, which is involved in assembling inflammasome complexes inside innate immunity cells [27, 28].

**Fig. 4:**
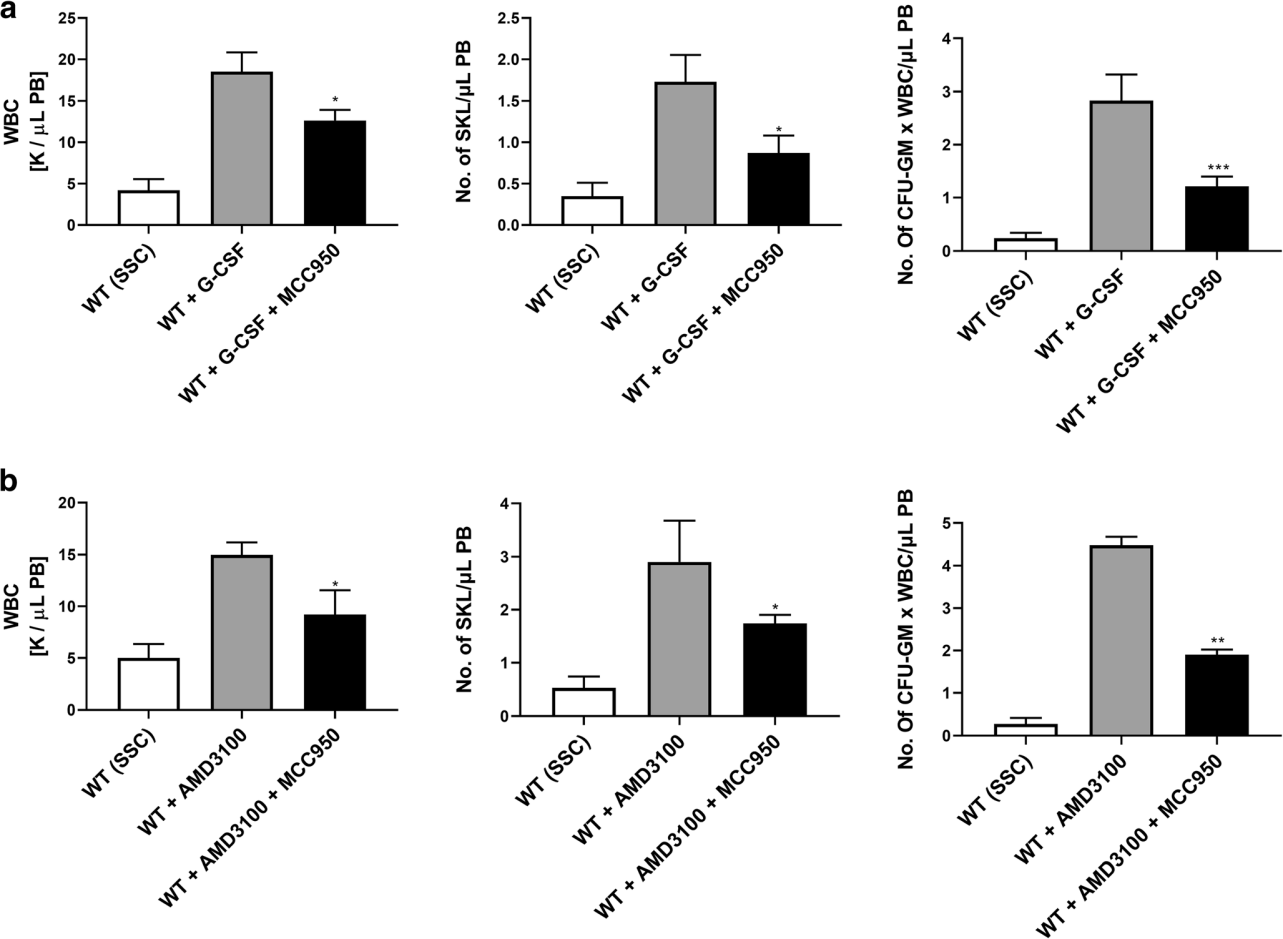

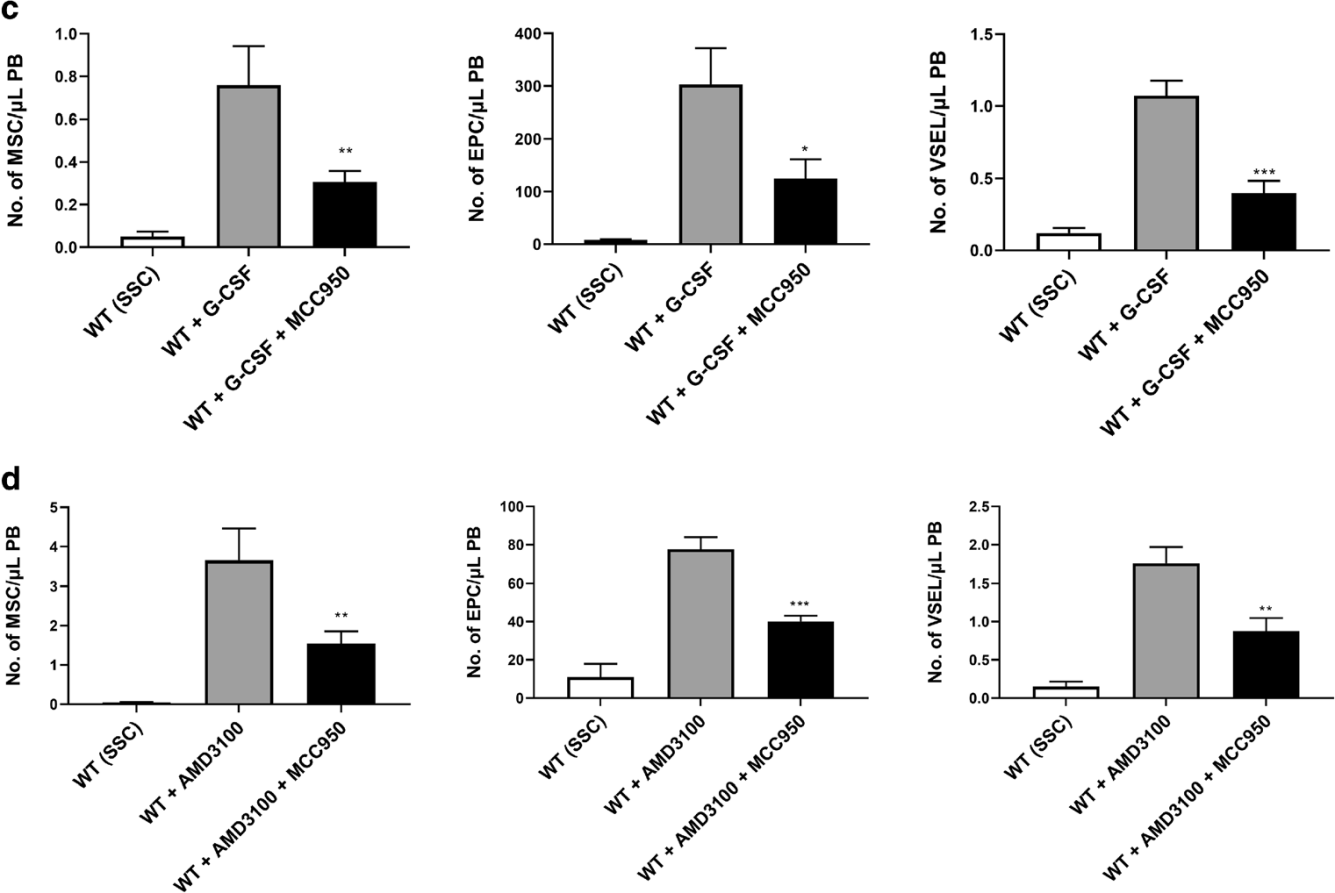
**Inhibition of the Nlrp3 inflammasome decreases mobilization of murine BM-residing stem cells.** Mononuclear cells (MNCs) were isolated from WT mice after 6 h following 3 days of G-CSF mobilization (Panels **a**, **c**) or 1 h after 1 dose of AMD3100 mobilization (Panels **b**, **d**), and treatment groups received additionally inflammasome inhibitor (MCC950) for 3 days. Panels **a–b**. The numbers of WBCs, SKL (Sca-1^+^/c-kit^+^/Lin^−^) cells, and CFU-GM clonogenic progenitors were evaluated in PB. WT (SSC) represents mice under steady-state conditions. Results from two independent experiments are pooled together. *p < 0.05; **p < 0.01; ***p < 0.001 comparing mobilized WT with mobilized WT administered with MCC950. Panels **c-d**. The numbers of MSCs (Lin^−^/CD45^−^/CD31^−^/CD90^+^), EPCs (Lin^−^/CD45^−^/CD31^+^), and VSELs (Sca-1^+^/Lin^−^/CD45^−^) in PB. WT (SSC) represents mice under steady-state conditions. Results from two independent experiments are pooled together. *p < 0.05; **p < 0.01; ***p < 0.001 comparing mobilized WT with mobilized WT administered with MCC950

As shown in Fig. 4a-b, inhibition of the Nlrp3 inflammasome in wild type (WT) mice exposed to G-CSF and AMD3100 resulted in significant inhibition of HSPC mobilization. Moreover, there was a visible decrease in mobilization of other types of BM-residing stem/progenitor cells, including MSCs and EPCs. Interestingly, we also observed a decrease in mobilization of the rare population of VSELs (Fig. 4c-d). In parallel, we observed a decrease in the levels of IL-1β, IL-18, Hmgb1, and C5a in PB by ELISA assay (data not shown). To validate data generated with the Nrlp3 inflammasome inhibitor MCC950, we performed G-CSF and AMD3100 mobilization in Nlrp3-KO mice and confirmed a significant decrease in egress of HSPCs from BM into PB. This decrease in mobilization was similar to that observed after administration of the inhibitor MCC950 (manuscript in preparation).

Since inhibition of HSPC mobilization was not complete, this finding suggests potential involvement and compensation by other members of the inflammasome family in this process, and we are currently investigating this possibility in our laboratories.

### Nlrp3 Inflammasome-Released Mediators Potentiate Mobilization of BM-Residing Stem Cells

Activation of the Nlrp3 inflammasome in cells from the innate immunity network leads to release of interleukin 1β and interleukin 18 as well as several DAMPs, including Hmgb1 and S1009a [17–20, 29]. As reported, IL-1β and IL-18 may act in an autocrine positive feedback-dependent manner to potentiate activation of the Nlrp3 inflammasome. On the other hand, DAMPs (Hmgb1 and S1009a) are recognized by MBL, which is a pattern-recognition receptor (PRR) circulating in PB. Binding of DAMPs to MBL leads to activation of mannan-associated serum proteases (MASPs), which trigger ComC activation in an MBL pathway-dependent manner [13, 29].

**Fig. 5:**
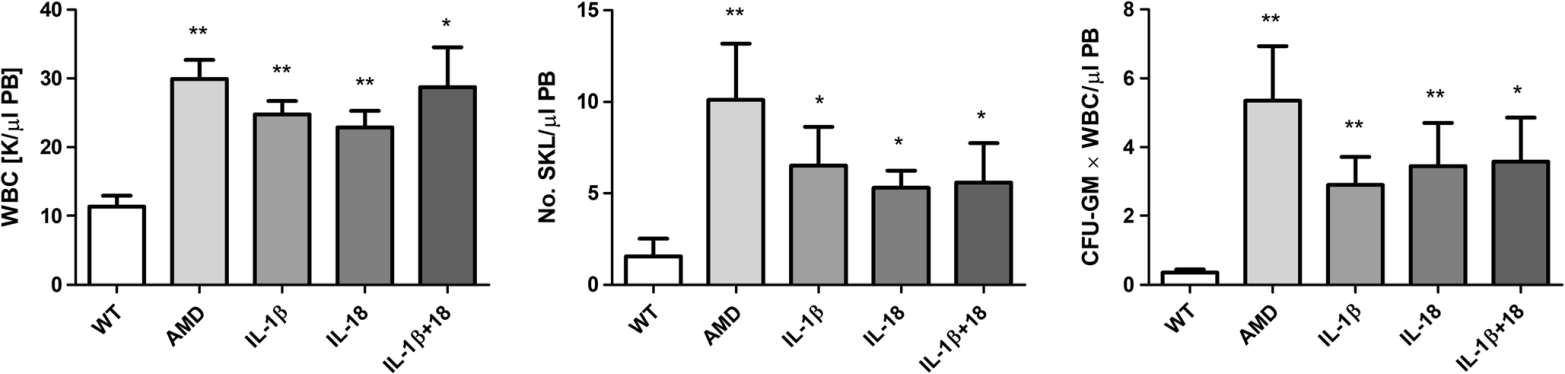

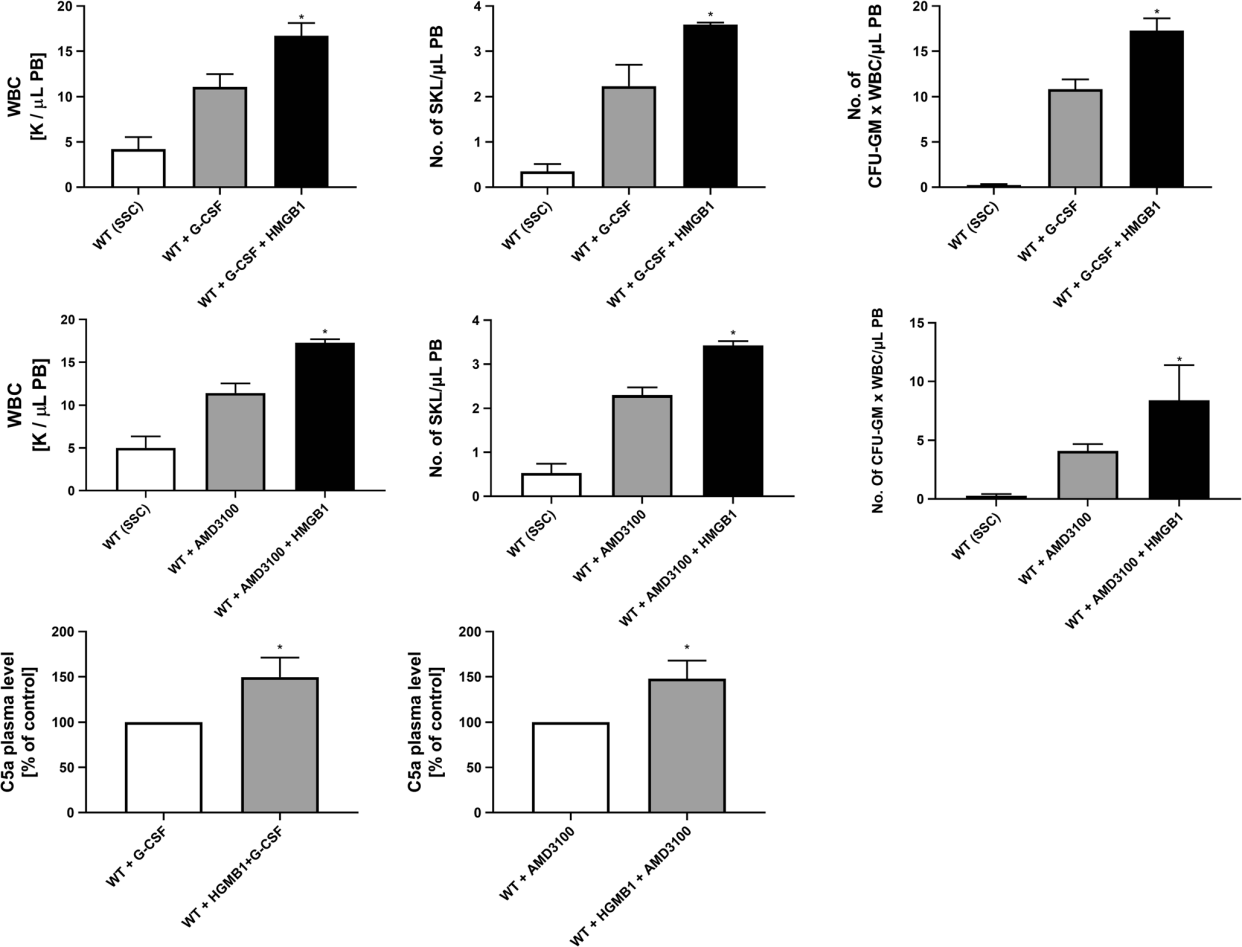
**Effect of Nlrp3 inflammasome activation on the release of factors affecting mobilization of HSPCs. Panel A. The interleukins IL-1β and IL-18 mobilize HSPCs**. Representative PB analysis for WBCs (left panel), SKL cells (middle panel), CFU-GM clonogenic progenitors (right panel, 3–4 independent experiments) from mice injected (IP) with PBS or AMD3100 (5 mg/kg), IL-1β alone or IL-18 alone (1 μg/mice), or with both interleukins (1 μg /mouse). AMD3100-injected mice were sacrificed 1 h post-injection, whereas interleukin-injected mice were sacrificed 6 h post last injection. The number of SKL cells mobilized in PB was calculated using the formula, WBCs x SKL cells/gated WBCs = SKL cells/μL; and, CFU-GM/μL of PB was evaluated by the formula, [WBCs] x CFU-GM colonies/WBCs plated. Data are presented as means ± S.E. Unpaired Student’s *t* test was used for the determination of significance (*, *p* ≤ 0.05, **, *p* ≤ 0.01). Panels **b–d.** Hmgb1 enhances G-CSF- and AMD300-directed mobilization of murine HSPCs. Mononuclear cells (MNCs) were isolated from WT mice after 6 h 3 days of G-CSF mobilization (Panel **b**) or 1 h after 1 dose of AMD3100 mobilization (Panel **c**), and the treatment groups received additionally HMGB1 for 3 days. The numbers of WBCs, SKL (Sca-1^+^/c-kit^+^/Lin^−^) cells, and CFU-GM clonogenic progenitors were evaluated in PB. WT (SSC) represents mice under steady-state conditions. Results from two independent experiments are pooled together. *p < 0.05; **p < 0.01; ***p < 0.001 compare mobilized WT with mobilized WT administered with HMGB1. Panel D. Activation of the complement cascade and release of C5a after G-CSF or AMD3100 mobilization together with HMGB1 administration. C5a level was measured in PB by employing a sensitive ELISA assay. *p < 0.05; **p < 0.01; ***p < 0.001 compared with control

On the other hand, IL-1β mobilizes HSPCs after in vivo administration and induces leukocytosis in mice [30]. To more directly address the role of IL-1β and IL-18 in the mobilization process, we performed in vivo experiments in WT mice using a single injection of IL-1β or IL-18 and compared their mobilization efficiency to a single injection of AMD3100 (Fig. 5a). We confirmed that injection of IL-1β alone promotes mobilization of HSPCs [30] and, what is more important, demonstrated for the first time that IL-18 has a similar effect. Interestingly, at the doses employed, IL-1β alone or IL-18 alone mobilized up to ~60% of the HSPCs as did AMD3100.

Moreover, since DAMPs (Hmgb1 and S1009a) are recognized by MBL, which subsequently activates mannan-associated serum proteases (MASPs) and thus triggers the MBL-dependent pathway of the ComC, we added Hmgb1 protein to injections of G-CSF (Fig. 5b) or AMD3100 (Fig. 5c). In both cases, addition of Hmgb1 protein enhanced mobilization efficacy in mice (Fig. 5b, c) and increased activation of the ComC, as measured by detection of the C5a cleavage fragment in PB (Fig. 5d).

## Discussion

Pharmacological mobilization is a means to obtain HSPCs for hematopoietic transplantation for clinical purposes and is induced by certain pro-mobilizing drugs, including G-CSF and AMD3100 [1, 6–10, 31–33]. During administration of these drugs the number of HSPCs in PB may increase by up to 100 fold over the steady-state level. Mobilized HSPCs are subsequently harvested from PB by leukapheresis. Our previous and current findings indicate the important role of purinergic signaling and innate immunity in this process [12, 13, 34], and the seminal observation of our current work is the observation that the ATP–Nlrp3 inflammasome–ComC axis orchestrates optimal egress of BM-residing stem cells into PB. This work also suggests postulated by us a novel role for the ATP-driven Nlrp3 inflammasome as a cogwheel or gear that connects purinergic signaling with activation of the ComC [29].

In support of such a mechanism, ATP has been reported to be a potent activator of the Nlrp3 inflammasome in several cell types, including hematopoietic cells, belonging to the innate immune system [17–20]. This effect occurs after ATP binding to the P2X7 purinergic receptor and involves influx of Ca^2+^ into cells as well as simultaneous efflux of K^+^ via the TWIK-2 potassium channel [25]. In our previous work we demonstrated that ATP is released from cells after stimulation by G-CSF or AMD3100 in a pannexin 1 channel-dependent manner [12, 13]. In support of this finding, we also found that ATP release induced by the pannexin 1-blocking drug probenecid or a pannexin 1-blocking peptide significantly decreased mobilization efficacy, and G-CSF-induced mobilization was impaired in P2X7 receptor-KO mice [12, 13]. To support this as mentioned above, the ATP–P2X7 interaction triggers activation of the Nlrp3 inflammasome [17–20, 25].

As demonstrated in our current work, Gr-1^+^/CD11b^+^ monocytes and granulocytes belonging to the innate immunity network activate the Nlrp3 inflammasome in response to ATP stimulation. Since G-CSF alone or AMD3100 alone were not able to do this, our results indicate the important role of ATP and purinergic signaling in the initial phase of mobilization, which first requires release of ATP from BM cells into the BM microenvironment in response to pro-mobilizing agents [12, 13]. This supports our previous finding that extracellular ATP is a trigger for the mobilization process and supports our hypothesis that the Nlrp3 inflammasome is a cogwheel or gear between ATP-directed purinergic signaling and activation of the ComC, which is required for egress of HSPCs from BM into PB [29].

The Nlrp3 inflammasome belongs to a broader family of inflammasomes and is a multiprotein oligomer responsible for activation of inflammatory responses [17–20]. It is expressed by myeloid cells, including Gr-1^+^ granulocytes and Gr-1^+^/CD11b^+^ monocytes, and is currently the best-characterized member of the inflammasome family operating in these cells. The Nlrp3 inflammasome is composed of several proteins, including NLRP3, ASC (Pycard), and caspase 1. Triggering of the Nlrp3 inflammasome activates intracellular caspase 1, which promotes maturation and secretion of pro-inflammatory cytokines, such as interleukin 1β (IL-1β) and interleukin 18 (IL-18), which are activated inside cells by proteolytic cleavage of their pro-forms (pro-IL-1 and pro-IL-18) before release into the extracellular space [17–20]. Caspase 1 also cleaves gasdermin protein, which releases N-gasdermin fragments that become subsequently inserted into cell membranes to from pores that facilitate release of IL-1β and IL-18 as well as important DAMPs, including the Hmgb1 and S100a9 proteins [20].

Here we demonstrate that the Nlrp3 inflammasome becomes activated at the mRNA and protein levels in BM and PB cells after administration of G-CSF or AMD3100. This activation was reflected by upregulation of mRNAs for Nlrp3, ASC (Pycard), caspase 1, IL-1β, IL-18, Hmgb1, and S1009a. In parallel, we observed an increase in IL-1β, IL-18, and Hmgb1 at the protein level in PB. In fact, an increase in the PB level of IL-1β and IL-18is one of the acknowledged indicators of Nlrp3 inflammasome activation [17–20].

Our results also indicate that IL-1β and IL-18 released from Nlrp3 inflammasome-expressing cells may on their own also promote egress of HSPCs from BM. IL-1β induces fever and leukocytosis in mice, and IL-1β has been demonstrated in the past to be a mobilizing cytokine [30]. In our work, for the first time we demonstrated that IL-18 has similar properties. In addition to both interleukins, the release of Hmgb1 and S1009a from cells triggers activation of the MBL-pathway of the ComC [13, 14].

We have demonstrated in the past that the MBL–ComC pathway is critical for optimal release of HSPCs from BM, and the distal ComC cleavage products, the C5a and _desArg_C5a anaphylatoxins as well as C5b-C9 (also known as the membrane attack complex, MAC), orchestrate optimal mobilization of HSPCs [24]. Here we confirm this finding based on the presence of anaphylatoxin C5a in PB, which indicates that the distal ComC is activated in response to DAMPs [23, 24].

Moreover, the DAMPs Hmgb1 and S100a9 are not only important activators of the ComC in the MBL-dependent pathway but in parallel also trigger activation of the coagulation cascade (CoaC) [8, 13, 35]. To explain this, DAMPs bind to MBL that subsequently triggers mannan-activated serine proteases (MASPs) to activate both ComC and in parallel also CoaC. As reported thrombin released during CoaC activation possesses C5-like convertase activity and facilitates, along with classical C5 convertase, cleavage of C5 and release of C5a and _desArg_C5a and thus plays a supportive role in HSPC mobilization [36, 37].

Our experiments with an Nlrp3 inflammasome stimulator (nigericin) and a small-molecule inhibitor (MC9950) confirmed its critical role in G-CSF- and AMD3100-induced mobilization. This role has also been supported by mobilization experiments in Nlrp3-KO mice. Further studies, however, are required to determine whether this defect is related to a lack of Nrlp3 in HSPCs or in cells in the BM microenvironment. It is also worthwhile mentioning that we also observed some differences in Nlrp3 inflammasome activation in the short-term (1-h) response to AMD3100 and G-CSF, which suggests some differences in the Nlrp3 inflammasome response to these different pro-mobilizing agents. These differences are currently being investigated in our laboratories. Furthermore, besides its role in the mobilization of HSPCs, the Nlrp3 inflammasome has several other pleiotropic effects and we are currently studying its role in the homing and engraftment of HSPCs after transplantation [29].

Interstingly, some recent results indicate that the AIM2 and NLRC4 inflammasomes may also be activated by factors released during “sterile” inflammation, including ATP and other DAMPs [38], and their potential involvement in HSPC mobilization requires clarification. Finally, further work should also address the role of NLRP family members (NLRP2, NLRC3, NLRP6, NLRP7, NLRP10, NLRP12, and NLRX1) in inhibiting inflammation, as they could have a negative role in the release of HSPCs from BM [17–20]. We can expect both positive and negative effects on mobilization from different inflammasome family members.

In conclusion, our work has identified for the first time the novel role of the ATP-driven Nlrp3 inflammasome in mobilization of HSPCs as well as other types of stem/progenitor cells residing in BM. These results also further support the role of innate immunity-induced “sterile” inflammation in BM as a trigger in the process of stem cell mobilization [12, 13, 39–41].
